# Generation of a Highly Efficient and Chemically Inducible Gene Expression System

**DOI:** 10.3390/genes17091142

**Published:** 2026-09-18

**Authors:** Yizhong Zhang, Linqiong Deng, Qing Li, Likun Lian

**Affiliations:** 1 School of Ecological Engineering, Guizhou University of Engineering Science, Bijie 551700, China; linqiongd@163.com (L.D.); liqingdream@163.com (Q.L.); 2 School of Mining Engineering, Guizhou University of Engineering Science, Bijie 551700, China; lianlikun@126.com

**Keywords:** *alc* gene switch, gene expression, ethanol induction method

## Abstract

Background: Chemically inducible gene expression systems provide precise control over the temporal and spatial expression of genes. They are powerful tools for analyzing gene function during plant development and can effectively avoid the issues associated with transgene expression driven by constitutive promoters. The *alc* gene expression system derived from *Aspergillus nidulans* is one of the most promising chemically inducible expression systems in plants, owing to its significant potential for both basic research and agricultural applications. Previous studies have demonstrated the utility of the *alc* system in several dicot species, but systematic optimisation of promoter architecture and induction methodology in *Arabidopsis* remains comparatively limited. Methods: We employed a multi-level study design, combining *Agrobacterium*-mediated genetic transformation, GUS histochemical staining, and GUS quantitative assays. First, different types of *alc* gene expression systems were constructed through codon optimization of the *GUS* and *alcR* genes, and by incorporating the CaMV 35S minimal promoter (min35S), the TMV omega sequence, the Kozak sequence, and specific binding sites for the AlcR transcription factor. Second, these distinct *alc* expression systems were individually transformed into *Arabidopsis thaliana* to obtain single T-DNA insertion homozygous transgenic plants. Third, ethanol was applied via root drench, foliar spray, or vapor induction, and the performance of different *alc* systems was evaluated based on GUS protein expression, to determine the optimal induction conditions and identify the most efficient ethanol-inducible gene expression system. Results: Four *alc* systems (Ves1–Ves4) were successfully developed. *GUS* gene expression driven by these systems was precisely regulated by ethanol and was dependent on induction time and the method of induction. Among the induction methods, ethanol vapor was the most effective, followed by root drench, whereas foliar spray was the least effective. Furthermore, Ves1 was identified as the most efficient *alc* expression system, Ves2 as an efficient *alc* expression system, Ves3 as a usable *alc* expression system, and Ves4 as potentially defective with limited practical application value. Conclusions: This work lays a foundation for further research on the efficient *alc* system and the exploration of its molecular mechanism underlying high expression efficiency. The most efficient Ves1 and the highly efficient Ves2 provide a clear basis for selection in subsequent applied research, and they are expected to play a greater role in plant functional genomics and agricultural biotechnology.

## 1. Introduction

Since the first successful genetically modified crop in 1983, transgenic technology has continuously evolved, with various promoters being applied in plant genetic engineering. In genetic engineering, precise control of gene expression—primarily regulated by promoters and also influenced by enhancers and transcription factors—is crucial for achieving desired outcomes [1,2]. To date, a variety of constitutive promoters have been successfully used in transgenic plants. Such as the cauliflower mosaic virus (CaMV) 35S promoter and the *Agrobacterium* nopaline synthase (NOS) promoter [3,4], the actin promoter [5], and the ubiquitin promoter [6,7] have been widely employed. Constitutive promoters drive the expression of target genes in all organs throughout the entire developmental process, effectively enhancing the expression levels of target genes. This characteristic has led to the widespread application of constitutive promoters in transgenic plants, laying a solid foundation for the establishment of transgenic plants and the study and identification of gene functions. However, it is understandable that the constitutive overexpression of certain transgenes in all tissues throughout development may be unnecessary or even have detrimental effects on the plant [8,9], as under normal conditions, such overexpression may compete for energy required for protein or RNA synthesis, which is also essential for plant growth [10]. Furthermore, this system is not suitable for studies requiring the restriction of target gene expression to specific organs or particular stages of plant development, nor does it allow for artificial regulation of target gene expression [11,12].

To enable temporal and spatial control of target gene expression, several promoters have been adopted in gene expression systems, including tissue-specific promoters [12,13,14,15], environment-responsive promoters [16,17,18], stress-inducible promoters [19,20,21], and wound-responsive promoters [22,23], etc. Although these gene expression systems perform well in some plants, they are far from satisfactory for gene function identification and plant genetic improvement, and the limited number of tissue-specific promoters restricts the analysis of several cell types or tissues [24]. Furthermore, due to the iterative mode of plant development, there is no absolute definition in different developmental stages. Therefore, these promoters do not have a temporally restricted expression patter in a strict sense [12,25]. Simultaneously, the regulatory mechanism of most tissue-specific promoters at the transcriptional level is still unclear, resulting in uncertainties when target genes are driven by them, which hinders the wide application of this kind of promoter [26]. Due to the variability and complexity of environmental factors and unpredictable wounding, both environment-responsive promoters and wound-inducible promoters driving target genes can produce unexpected outcomes. This makes it difficult to precisely regulate the expression of exogenous target genes using these two types of promoters, and also limits their application in gene function studies and plant genetic improvement [26].

Compared with tissue-specific expression systems, environment-responsive expression systems, stress-inducible expression systems, and wound-responsive expression systems, chemical-inducible gene expression systems enable both temporal and spatial regulation of gene expression by artificial control, and can be used to more precisely studied gene function in plants [27,28]. Chemical-inducible expression system can induce the expression of the target gene at the desired time point, and the expression level can be easily controlled by changing the concentration of the inducer, the problems that may be related to constitutive overexpression can be avoided, and the main molecular events caused by the activation of specific genes can be revealed [13,29]. Therefore, the system is easy to be applied to various research, industrial fields and agricultural fields [13]. To date, scientists have successfully developed a series of chemical-inducible gene expression systems, including the tetracycline-inducible system [30,31], steroid-inducible system [32,33,34], ecdysone-inducible system [35,36], copper-inducible system [37,38], and ethanol-inducible system [39,40,41,42]. These systems have been widely applied in various research fields such as gene function analysis [43], marker-free transformation systems [44], site-specific excision [45], and RNA silencing [46,47,48], etc., providing powerful tools for basic research in plant biology and biotechnological applications. Among them, ethanol-inducible expression system has been considered as the most likely chemical-inducible system for field application because of its high induction efficiency, low background level, low cost, flexible use, and environmental safety [24,26]. The system consists of two components. The first one is the expression cassette of produced transcription factor AlcR (CaMV35S promoter/tissue-specific promoter: *alcR*), which is composed of CaMV35S promoter or tissue-specific promoter and *alcR* gene. The other one is target gene expression cassette (p*alcA*: min35S: target gene), which is composed of the upstream activator region of the *alcA* promoter (p*alcA*) and a 35S minimal promoter as well as a target gene. The active transcription factor AlcR binds to specific sites in the *alcA* promoter to drive target gene expression [12,27,42]. The mode of action of this system has been validated in *Arabidopsis* [39,43], tobacco [29,49,50], poplar [40], *Catharanthus roseus* [51], tomato [52], potato [29], sugarcane [41], and algae [42], etc. Meanwhile, studies have used this system to drive *alcR* expression under the control of the promoter of the floral meristem identity gene *LEAFY*, achieving flower-specific, ethanol-inducible gene expression in *Arabidopsis* [28].

Although these studies have enhanced our understanding of the ethanol-inducible expression system (alcR/alcA) and laid a foundation for gene expression regulation, the constructs used in them were mostly generated by fusing only the 35S minimal promoter sequence (min35S) downstream of the *alcA* promoter. These studies rarely involve the integration of enhancer sequences, Kozak sequences, the specific binding site sequence for the action of the AlcR transcription factor, and few studies have addressed codon optimization of the *alcR* gene and the *GUS* reporter gene. In addition, there is currently a lack of comparative studies among target genes driven by the alcR/alcA system, those driven by the 35S promoter, and those driven by the native *alcA* promoter. Therefore, we report an efficient *alc* gene switch developed through multilevel optimization in *A. thaliana*. Transgenic plants harboring this improved *alc* gene expression system displayed stable, ethanol-inducible gene expression. The improved *alc* gene switch was effectively induced in soil-grown plants through ethanol root drench, foliar spray, and vapor induction. The development of a highly efficient and chemically inducible gene expression system holds significant theoretical and practical value for the study of plant gene function and the real-time control of target gene expression, and may enhance its application in basic plant biology research and crop genetic improvement.

## 2. Materials and Methods

### 2.1. Vector Construction

**Figure 1:**
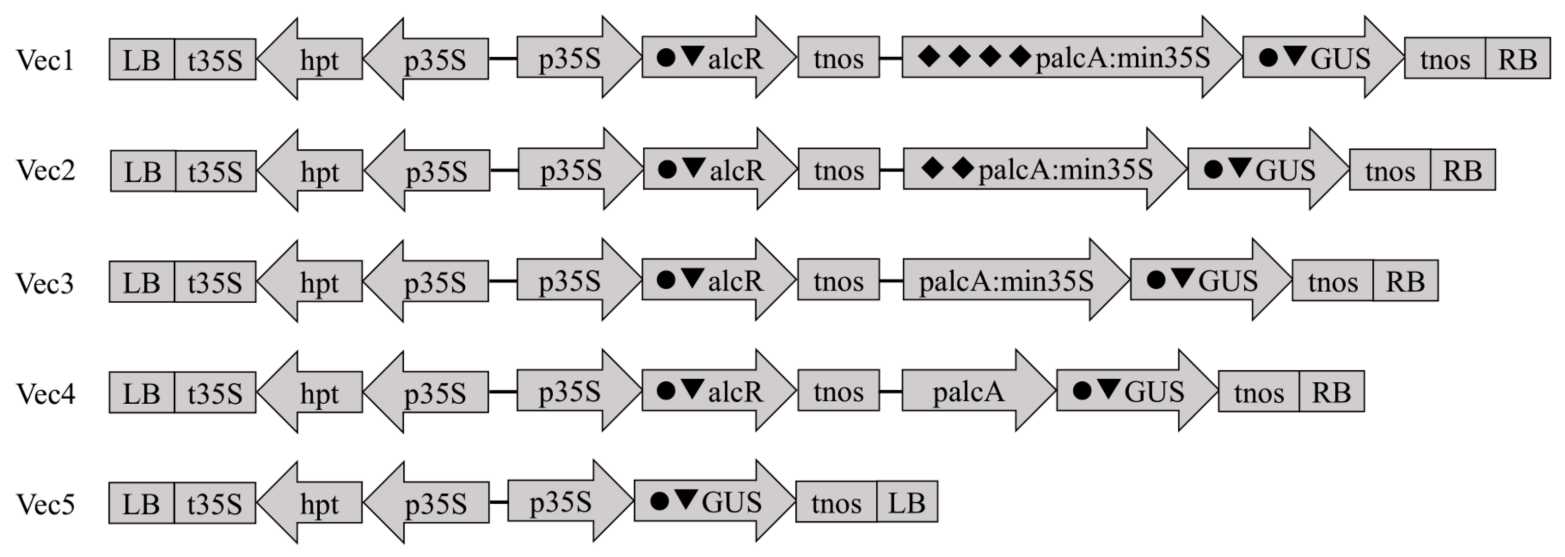
Schematic diagram of the *alcA* constructs. For Vec1, Vec2, Vec3, and Vec4, p35S: *hpt* and p35S: *alcR* both use the CaMV 35S promoter to express HPT protein and AlcR protein from their respective CDSs. The *alcA* reporter cassette, p*alcA*: min35S: *GUS*, contains a fusion promoter using the AlcR binding sites of the *alcA* promoter with the sequences downstream of the TATA sequence from the CaMV 35S promoter, to direct expression of the *GUS* reporter gene. For Vec5, p35S: *hpt* and p35S: *GUS* both use the CaMV 35S promoter to express HPT protein and GUS protein from their respective CDSs. ●, ▼, and ◆ represent translational enhancer sequence from the tobacco mosaic virus (TMV) (tatttttacaacaattaccaacaacaacaaacaacaaacaacattacaattactatttacaattaca), Kozak sequence (gcggccgcc), and the specific binding site sequences for the action of the AlcR protein (atgcatgcggaaccgcacgagg), respectively.

According to the literature [27,53,54,55], the sequences of the cauliflower mosaic virus 35S (CaMV 35S) promoter sequence, the 35S minimal promoter sequence (min35S), the *alcA* promoter sequence, the *Agrobacterium* nopaline synthase (NOS) terminator sequence, the *alcR* gene coding sequence, the specific binding site sequence for the action of the AlcR transcription factor, the *GUS* (*β*-glucuronidase) gene coding sequence, the enhancer sequence, and the Kozak sequence were retrieved from the National Center for Biotechnology Information (NCBI) (http://www.ncbi.nlm.nih.gov). Restriction enzyme site analysis of the *GUS* and *alcR* gene nucleotide sequences was performed using DNAMAN software(9.0), while the pCAMBIA1300 vector sequence was analyzed with SnapGene software to identify suitable restriction sites. The details are as follows: (1) Rare codons in the *alcR* and *GUS* genes were identified using the GenScript website (https://www.genscript.com.cn/tools/rare-codon-analysis) (accessed on 6 March 2026) and DETAIBIO website (https://www.detaibio.com/contact-us.html) (accessed on 6 March 2026) based on the codon usage bias of *A. thaliana*, without altering the encoded protein sequences, the codons were modified to those preferred by *A. thaliana*. (2) The translational enhancer sequence from the tobacco mosaic virus (TMV) followed by an alternative Kozak sequence were placed immediately upstream of the *alcR* and *GUS* coding sequences. (3) Different numbers of specific binding site sequences for the action of the AlcR protein and the CaMV 35S minimal promoter sequence (min35S) were integrated upstream and downstream of the *alcA* promoter, respectively. (4) *GUS* gene expression driven by the CaMV35S promoter as a control of the a high-efficient *alc* gene switch. (5) Based on the structure of the pCAMBIA1300 vector, restriction enzyme recognition sites were placed at the corresponding positions of each gene expression cassette. The various sequences were assembled and synthesized, and subsequently cloned into the pCAMBIA1300 vector. This process yielded different types of vectors for alcR/alcA-driven or 35S-driven expression of the *GUS* reporter gene: pCA13alcRalcA1-*GUS* (Vec1), pCA13alcRalcA2-*GUS* (Vec2), pCA13alcRalcA3-*GUS* (Vec3), pCA13alcRalcA4-*GUS* (Vec4), and pCA13-35-*GUS* (Vec5). The T-DNA regions of each vector are listed in Figure 1.

### 2.2. Plant Transformation, Growth, and Maintenance

The Columbia ecotype (wild type, WT) of *A. thaliana* used for genetic transformation was maintained by our laboratory. *Agrobacterium* GV3101 cells harboring the recombinant plasmid were selected, and a single colony was inoculated into 50 mL of YEB medium (containing 50 μL Kan, 50 μL Rif, and 25 μL Str) and cultured at 28 °C with shaking at 230 rpm until OD_600_ reached 0.7 (approximately 24 h). The bacterial cells were collected and resuspended in infiltration solution to an OD_600_ of 0.6, followed by the addition of Silwet L-77 to a final concentration of 0.04%. *A. thaliana* plants grown in a nutrient soil and vermiculite mixture (3:1, *v*/*v*) in pots (length × width × height = 7 × 7 × 8 cm) had all open flowers and mature siliques removed, and the inflorescences were immersed in the infiltration solution for 30 s. The plants were then placed horizontally in plastic boxes (length × width × height = 53 cm × 39 cm × 32 cm) and incubated in the dark for 24 h, after which they were transferred to a light incubator (12,000 Lux, 23.0 °C, 16 h light; 0 Lux, 22.0 °C, 8 h dark) until the seeds matured. Seeds (T0) were harvested, dried, and stored at 4 °C.

### 2.3. Screening for Transgenic Homozygous Plants with a Single T-DNA Insertion

T0 seeds were surface-sterilized with 5.0% NaClO solution for 10 min, thoroughly rinsed with sterile water, and sown on MS medium containing hygromycin. After stratification at 4 °C for 55 h, the plates were transferred to a light incubator under the following conditions: 12,000 Lux, 23.0 °C, 16 h light; 0 Lux, 22.0 °C, 8 h dark. Resistant seedlings were transplanted into pots (length × width × height = 7 cm × 7 cm × 8 cm), and transgenic plants were confirmed by PCR using *GUS*-specific primers (Thermo Fisher Scientific, Waltham, MA, USA) (forward: 5′- cgccatgcttagacctgttg-3′; reverse: 5′- cattgtttacctccttgttgagg-3′) and GUS staining. The PCR cycling conditions were: 94 °C for 3 min; 40 cycles of 94 °C for 30 s, 59 °C for 30 s, and 72 °C for 2 min; and a final extension at 72 °C for 5 min. These plants were then self-pollinated, and seeds (T1) were collected from individual plants. After drying, 30 seeds from a single T1 plant were processed and cultured using the same method as described above. Following PCR and GUS staining identification, the numbers of transgenic and non-transgenic plants were counted. A chi-square test was performed to determine the inheritance pattern of the transgene. Upon maturity, seeds (T2) were collected from individual plants. The process of sowing, screening, and collecting seeds from single plants was repeated until homozygous transgenic plants were obtained. Ultimately, homozygous transformants carrying a single T-DNA insertion for each vector construct were obtained.

### 2.4. Ethanol Treatment

Three-week-old soil-grown *A. thaliana* seedlings in pots (length × width × height = 7 cm × 7 cm × 8 cm) were placed in a transparent plastic container (length × width × height = 53 cm × 39 cm × 32 cm). Ethanol treatments were applied as follows: 1.5% ethanol (*v*/*v*) was used for root drench (12 mL per pot), 5.0% ethanol (*v*/*v*) was applied as a foliar spray until droplets run off, and 5.5 mL of 95% ethanol (*v*/*v*) was put into a 10 mL glass beaker, which was then positioned in one corner of a sealed transparent plastic container and was used for vapor induction (the lid was removed after 96 h). The seedlings were then cultured in a light incubator under the following conditions: 12,000 Lux, 23.0 °C, 16 h light; 0 Lux, 22.0 °C, 8 h dark. Leaf samples (0.1 g each) were collected at 12 h, 24 h, and 48 h after treatment for GUS protein.

### 2.5. Analysis of GUS Accumulation

Protein was extracted from the leaves of three-week-old plants by using the method described [56]. Extraction solution contains 50 mM Na_3_PO_4_, 10 mM Na_2_EDTA, 1.6 mM TritonX-100, 3.4 mM sarcosyl, and 10 mM β-Mercaptoethanol. The total soluble protein was determined as described by Modified Bradford Protein Assay Kit (Sangon, Shanghai, China). For histochemical analysis, the GUS activity was determined as described by GUS Staining Kit (Coolaber, Beijing, China). The young leaves and the flower buds were soaked in GUS staining solution and incubated at 37 °C in the dark for 6 h. After discarding the staining solution, decolorization was performed four times with 75.0% ethanol, each time for 1 h, until the negative control materials were completely decolorized. For quantitative analysis, a fluorimetric assay was conducted using 4-methylumbelliferyl β-D-glucuronide as a substrate. GUS activity was quantified using a Microplate Reader (Tecan Company, Männedorf, Switzerland) at excitation and emission wavelengths of 365 nm and 455 nm, respectively. Quantification was performed using 100 mM 4-methylumbelliferone (4MU) as a standard.

### 2.6. Analysis of Agronomic Traits and Physiological Indexes

Three-week-old soil-grown *A. thaliana* seedlings in pots (length × width × height = 7 cm × 7 cm × 8 cm) were placed in a transparent plastic container (length × width × height = 53 cm × 39 cm × 32 cm), and were subjected to root drench with 1.5% ethanol for 48 h. Chlorophyll content, superoxide dismutase (SOD), peroxidase (POD), and catalase (CAT) activities were determined [57,58]. When the seeds were mature, the plant height and seeds per silique were analyzed.

### 2.7. Statistical Analysis

A one-way analysis of variance was performed to determine the differences.

## 3. Results

### 3.1. Construction and Testing of Inducible Plant Gene Expression Vectors

To generate a highly efficient *alc* gene switch in *A. thaliana*, we designed and tested four different *alcA* promoter vectors and one 35S promoter vector as a control (Figure 1; Supplemental Table S1). The specific protocol was as follows: (1) The rare codons in the *alcR* and *GUS* genes were identified and modified using the GenScript and DETAIBIO websites, as these rare codons have been demonstrated to significantly reduce translation rates and the yield of the encoded proteins [59]. (2) A 35S minimal promoter containing only those sequences between positions −31 and +3, which lacks the ability to initiate transcription, was fused downstream of the *alcA* promoter, and the resulting construct was verified to substantially increase chemical-inducible gene expression in *Nicotiana tabacum* [60,61]. (3) It was verified that the fusion of the entire upstream region of the *A. nidulans alcA* promoter, which contains an additional AlcR binding site, is important for ethanol inducibility [62]. (4) In the *alc* gene switch, fusing different numbers of tandem AlcR inverted repeat binding sites—which have been shown to enhance protein expression levels—leads to higher transcriptional activity [41]. (5) Incorporation of the Kozak sequence into eukaryotic gene expression vectors can improve protein expression levels in cells without affecting the properties or functions of the target protein [63]. (6) Fusion of the translational enhancer sequence of the tobacco mosaic virus (TMV) omega element, placed immediately upstream of the *alcR* and *GUS* coding sequences, could increase the translational efficiency of plant mRNAs to which it was fused by 1.5- to 3-fold [64,65].

To obtain transgenic *A. thaliana* lines with a homozygous single T-DNA insertion, positive seedlings were selected on hygromycin-containing medium and subsequently transplanted to soil. Then the plants were self-pollinated, and progeny were subject to segregation analysis for hygromycin resistance. Single T-DNA insertion plants transformed (hygR: hygS = 3:1 *p* > 0.30) with Vec1, Vec2, Vec3, Vec4, and Vec5 vectors were obtained, with 115, 112, 96, 104, and 95 plants, respectively. Subsequently, homozygous single T-DNA insertion transgenic lines were obtained (Supplemental Table S2).

**Figure 2:**
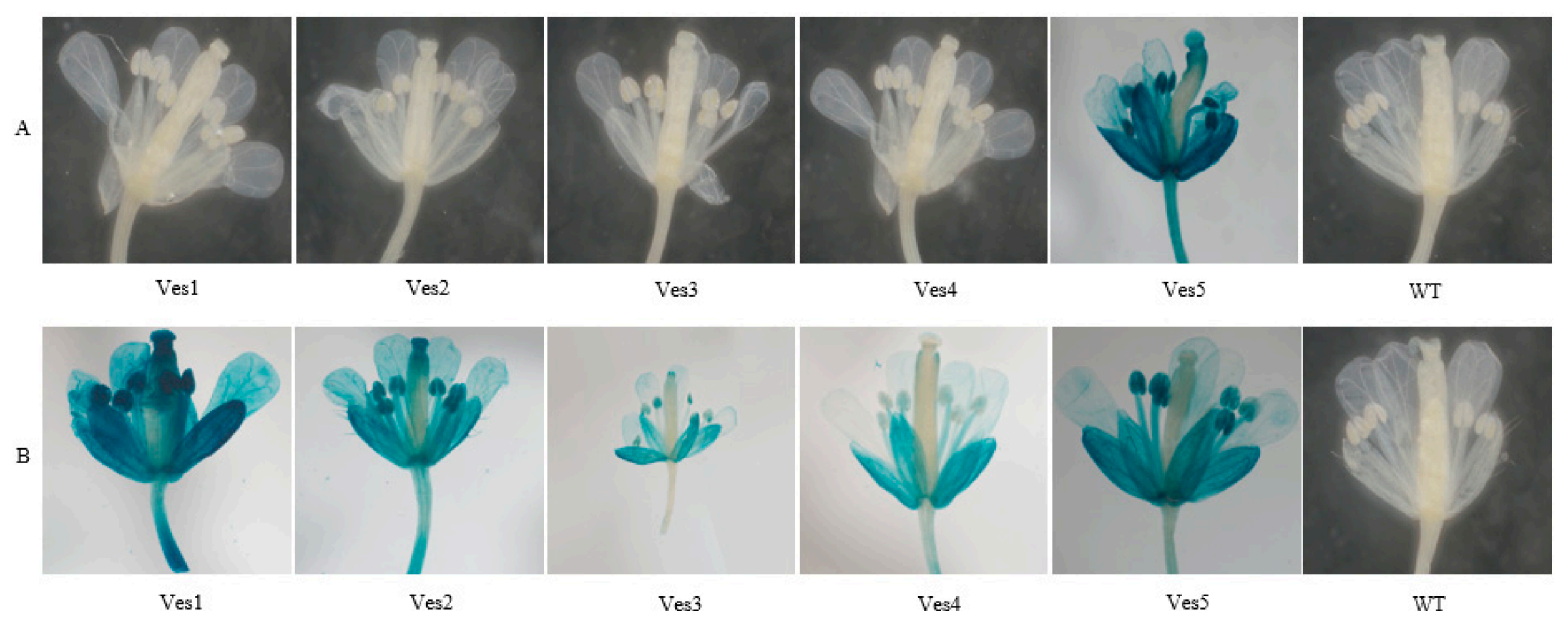
Ethanol-inducible GUS expression from the *alc* system in transgenic *A. thaliana*. Histochemical staining of flower buds for GUS activity was conducted using three-week-old soil-grown single T-DNA insertion homozygous seedlings containing different *alc* systems. Induction was achieved by root drench with 1.5% ethanol or distilled water for 16 h before analysis. A wild-type (WT) plant and a constitutive 35S-GUS plant were used as negative and positive controls, respectively. (**A**,**B**) were treated with distilled water and ethanol, respectively.

**Figure 3:**
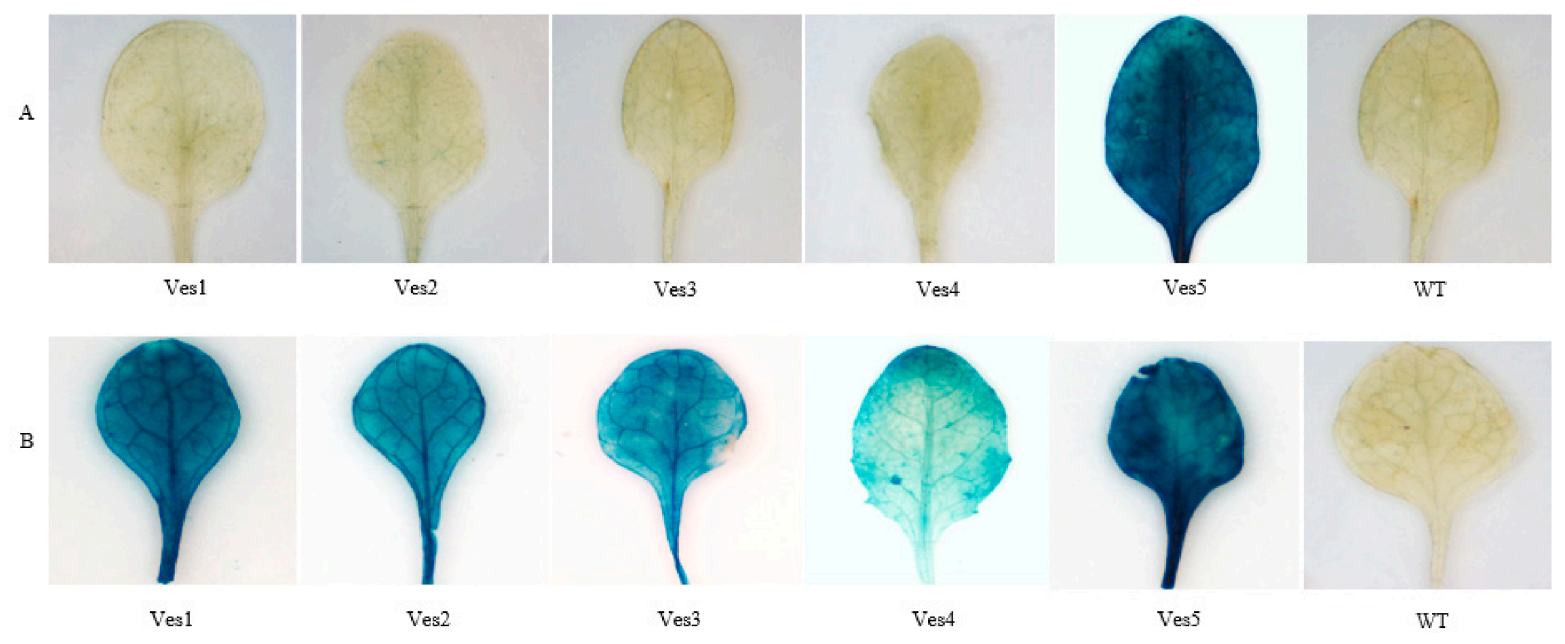
Ethanol-inducible GUS expression from the *alc* system in transgenic *A. thaliana*. Histochemical staining of young leaves for GUS activity was conducted using three-week-old soil-grown single T-DNA insertion homozygous seedlings containing different *alc* systems. Induction was achieved by root drench with 1.5% ethanol or distilled water for 16 h before analysis. A wild-type (WT) plant and a constitutive 35S-GUS plant were used as negative and positive controls, respectively. (**A**,**B**) were treated with distilled water and ethanol, respectively.

Ethanol-inducible *GUS* gene expression was further studied using GUS histochemical staining of the leaves and the flower buds from three-week-old soil-grown *A. thaliana* seedlings treated with 1.5% ethanol via root drench for 16 h. The results showed that when treated with distilled water, no GUS protein was produced in the stigmas, styles, stamens, petals, leaves, and other tissues of plants containing Ves1, Ves2, Ves3, or Ves4, nor in WT plants (Ves1–Ves4 and WT in Figure 2A and Figure 3A). In contrast, when treated with a 1.5% ethanol, GUS protein was produced in the stigmas, styles, stamens, petals, leaves, and other tissues of plants containing Ves1, Ves2, Ves3, or Ves4 (Ves1–Ves4 in Figure 2B and Figure 3B), but no GUS protein was detected in WT plants (WT in Figure 2 and Figure 3). Plants containing Ves5 produced GUS protein regardless of whether they were treated with distilled water or a 1.5% ethanol (Ves5 in Figure 2 and Figure 3). These results indicate that *GUS* gene expression in plants containing Ves1, Ves2, Ves3, or Ves4 is precisely regulated by ethanol, whereas neither the plants containing Ves5 nor the wild-type plants exhibit this characteristic.

### 3.2. Comparisons Between the Inducible alc Expression and the CaMV35S Expression

**Figure 4:**
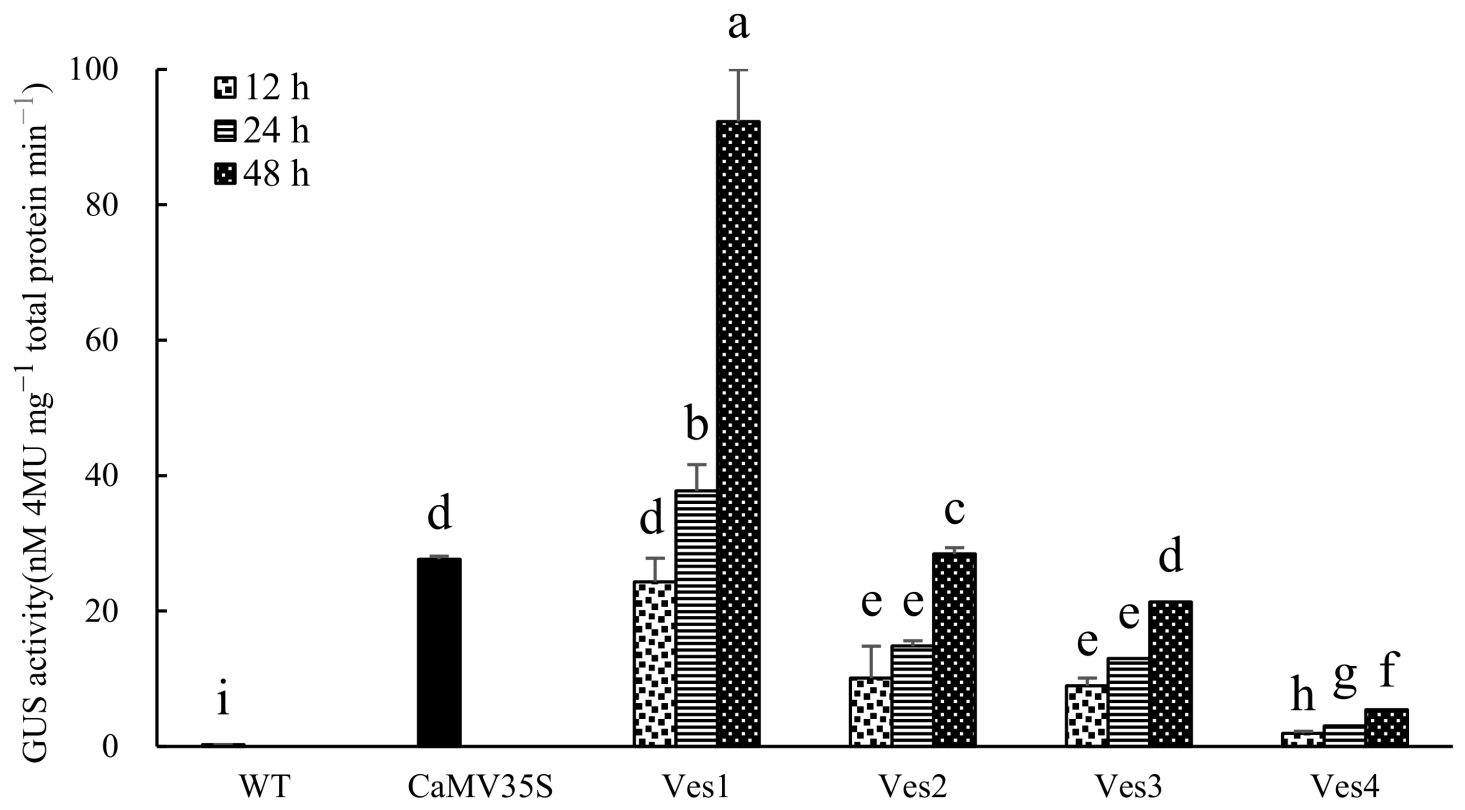
Comparison of constitutive and ethanol-inducible expression in transgenic *A. thaliana*. GUS expression level in leaves was analyzed using 3-week-old wild-type (WT) plants and transgenic plants containing a single T-DNA insertion of the 35S-GUS or *alcA*-GUS construct. Mean GUS expression level was shown for wild-type plants and 35S-GUS plants as well as four independent *alcA*-GUS plants at various times after treatment with 1.5% ethanol root drench. Data are mean ± SD. Different letters on the column chart indicate significant differences (*p* < 0.05).

To test the efficiency of the inducible *alc* expression system, the CaMV35S promoter driving the same *GUS* gene was used as a reference. Ethanol induction was performed via root drench, foliar spray, and ethanol vapor, and GUS accumulation was examined at different induction times. For the 1.5% ethanol root drench, the CaMV35S expression system was not affected by ethanol, while the *alc* expression systems (Ves1–Ves4) were regulated by ethanol induction, with significant variations observed. Ves1 achieved a relatively high GUS expression level of 24.2991 at the early induction stage (12 h), which was slightly lower than that of CaMV35S expression system (27.6552) with no significant difference, but significantly higher than that of Ves2 and Ves3 at both 12 h and 24 h after induction. At 24 h after induction, an even higher GUS expression level of 37.7278 was observed, which was 1.36-fold that of the CaMV35S expression system and significantly exceeded the CaMV35S level. By 48 h after induction, the GUS expression level increased rapidly to 92.2728, representing a 3.34-fold increase over the CaMV35S expression system and showing a significant advantage over it (Figure 4). For Ves2 and Ves3, GUS expression levels were relatively low at both 12 h and 24 h after induction and were significantly lower than those of CaMV35S. At 48 h after induction, Ves2 showed significantly higher expression than CaMV35S, while Ves3 exhibited no significant difference compared to CaMV35S. In contrast, Ves4 demonstrated consistently low GUS expression levels, which were significantly lower than those of the other four constructed vectors. The results indicated that the *alc* systems displayed a gradient in performance under 1.5% ethanol root drench treatment (Ves1 > Ves2 > Ves3 > Ves4). Consequently, Ves1 and Ves2 were determined to be the most efficient and highly efficient chemical induction systems, respectively.

**Figure 5:**
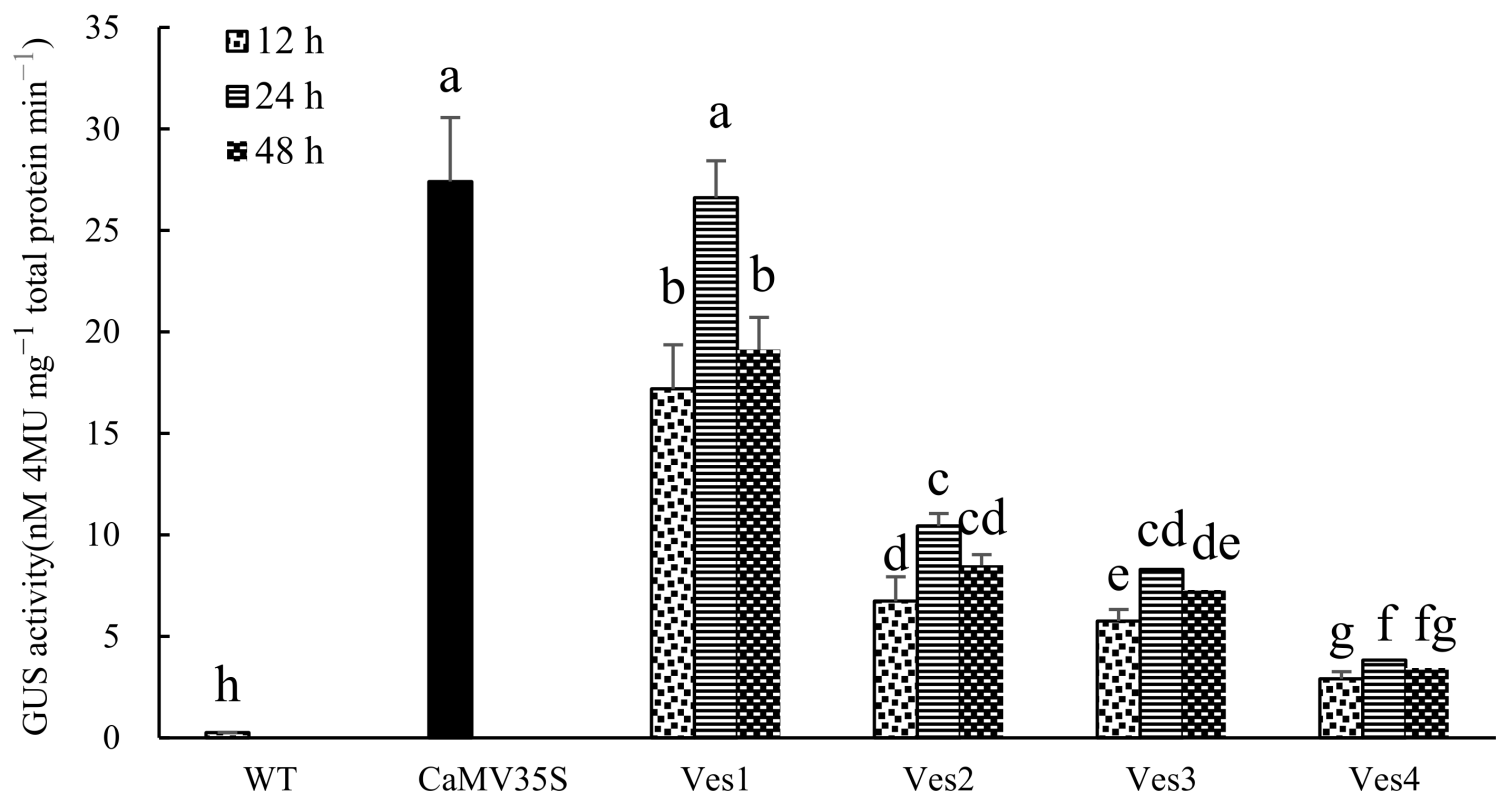
Comparison of constitutive and ethanol-inducible expression in transgenic *A. thaliana*. GUS expression level in leaves was analyzed using 3-week-old wild-type (WT) plants and transgenic plants containing a single T-DNA insertion of the 35S-GUS or *alcA*-GUS construct. Mean GUS expression level was shown for wild-type plants and 35S-GUS plants as well as four independent *alcA*-GUS plants at various times after treatment with 5.0% ethanol foliar spray. Data are mean ± SD. Different letters on the column chart indicate significant differences (*p* < 0.05).

For the 5.0% ethanol foliar spray, ethanol had no effect on the CaMV35S system, while it significantly regulated the *alc* systems (Ves1–Ves4). GUS expression levels of Ves1 at 12 h and 48 h after induction were 17.1952 and 19.1231, respectively, both significantly lower than that of the CaMV35S expression system (27.4117). At 24 h after induction, its GUS expression (26.6142) was slightly lower than that of the CaMV35S system, but the difference was not significant. In contrast, Ves2–Ves4 showed relatively low GUS expression levels at 12 h, 24 h, and 48 h after induction, all significantly lower than the CaMV35S system (Figure 5). The results indicated that the *alc* systems exhibited a gradient in performance under 5.0% ethanol foliar spray (Ves1 > Ves2 > Ves3 > Ves4). Therefore, Ves1 can serve as a relatively efficient chemically inducible system.

**Figure 6:**
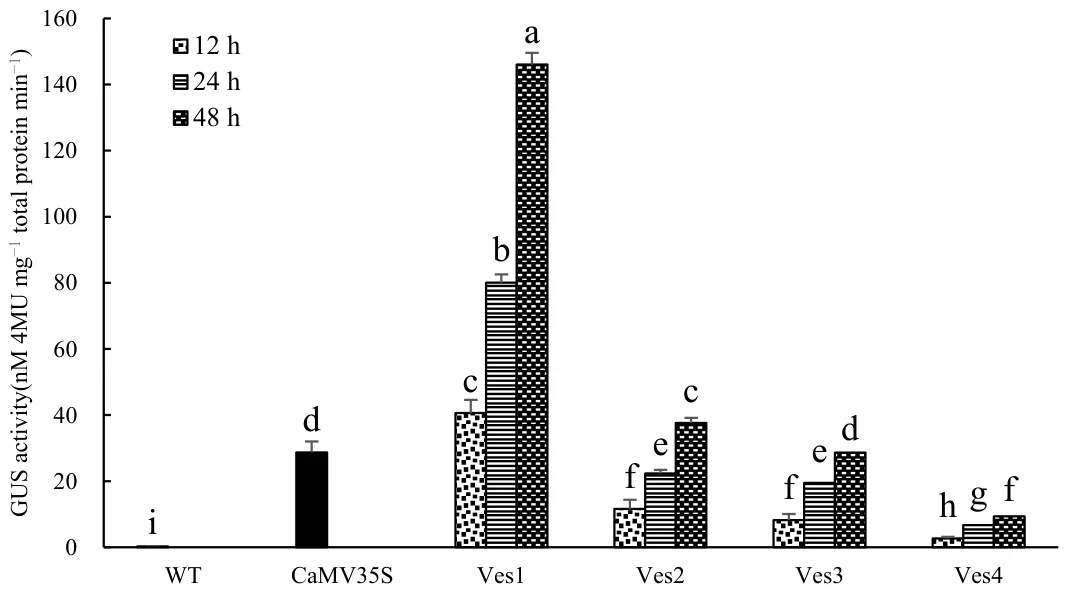
Comparison of constitutive and ethanol-inducible expression in transgenic *A. thaliana*. GUS expression level in leaves were analyzed using 3-week-old wild-type (WT) plants and transgenic plants containing a single T-DNA insertion of the 35S-GUS or *alcA*-GUS construct. Mean GUS expression level was shown for wild-type plants and 35S-GUS plants as well as four independent *alcA*-GUS plants at various times after treatment with 95% ethanol vapor induction. Data are mean ± SD. Different letters on the column chart indicate significant differences (*p* < 0.05).

For the 95% ethanol vapor induction, the CaMV35S expression system was not affected by ethanol, while the *alc* expression systems (Ves1–Ves4) were regulated by ethanol induction, with significant variations observed. Ves1 could obtain a higher GUS expression level of 40.6154 within a short induction time (12 h), which was 1.41-fold that of CaMV35S (27.6552) with a significant difference. Moreover, it also was significantly higher than the expression levels of Ves2 and Ves3 at 12 h and 24 h after induction. At 24 h after induction, an even higher GUS expression level of 80.0552 was observed, which was 2.79-fold that of the CaMV35S expression system and significantly exceeded the CaMV35S level. By 48 h after induction, the GUS expression level increased rapidly to 146.0380, representing a 5.08-fold increase over the CaMV35S expression system and showing a significant advantage over it (Figure 6). For Ves2 and Ves3, GUS expression levels were relatively low at both 12 h and 24 h after induction and were significantly lower than that of CaMV35S. At 48 h after induction, Ves2 and Ves3 were significantly higher than and had no significant difference with CaMV35S, respectively. In contrast, even after a long time of ethanol induction (24 h and 48 h), Ves4 still showed a persistently lower GUS expression level, which was significantly lower than that of the other four constructed vectors. The results indicated that under 95% ethanol vapor induction the GUS gene expression levels of the *alc* systems were sorted as: Ves1 > Ves2 > Ves3 > Ves4. Consequently, Ves1 and Ves2 were determined to be the most efficient and highly efficient chemical induction systems, respectively.

### 3.3. Efficiency Analysis of the alc System

To define the efficiency of the different Ves systems (Ves1–Ves4), we analyzed the induction fold and induction speed of GUS expression levels under different treatment methods. For the 1.5% ethanol root drench, in terms of induction fold, the GUS protein expression level of Ves1 12 h after induction was 55.54 times that of the uninduced control, and 86.23 times 24 h after induction. The induction folds at both time points were much higher than those of Ves2–Ves4, and the 24 h induction fold was 2.51 times that of Ves2. In terms of induction speed, a strong induction of 55.54-fold was achieved from 0 h to 12 h, accounting for 64.4% of the 24 h induction amount, indicating an extremely rapid response. Therefore, Ves1 is the most efficient *alc* induction system. For Ves2, the GUS protein expression level 12 h after induction was 23.39 times that of the uninduced control, and 34.39 times 24 h after induction. The induction folds at both time points were higher than those of Ves3 and Ves4. In terms of induction speed, a high induction of 23.39-fold was already achieved from 0 h to 12 h, accounting for 68.0% of the 24 h induction amount (Table 1), with a startup speed slightly faster than that of Ves1. Thus, Ves2 is an efficient *alc* induction system.

For the 5.0% ethanol foliar spray, Ves1 showed the best performance in terms of induction fold (at 12 h after induction, the GUS protein expression level was 44.04 times that of the uninduced control, and 68.16 times at 24 h after induction), much higher than those of Ves2–Ves4; its induction speed was such that 64.6% of the 24 h induction amount was already reached at 12 h after induction. Considering the expression advantages at both 12 h and 24 h after induction, Ves1 was defined as the most efficient *alc* induction system. Ves2 showed relatively good induction folds (at 12 h after induction, the GUS protein expression level was 17.96 times that of the uninduced control, and 27.85 times at 24 h after induction). Its induction speed (64.5% of the 24 h induction amount already reached at 12 h after induction) was consistent with that of Ves1, and its overall performance was better than those of Ves3 and Ves4 (Table 2), so it was defined as an efficient *alc* induction system.

For the 95% ethanol vapor induction, in terms of induction fold, the GUS protein expression level of Ves1 at 12 h after induction was 95.45 times that of the uninduced control, and 188.14 times at 24 h after induction. The induction folds at both time points were much higher than those of Ves2–Ves4, and the 24 h induction fold was 3.4 times that of Ves2. In terms of induction speed, a strong induction of 95.45-fold was already achieved from 0 h to 12 h, accounting for 50.7% of the 24 h induction amount, making it the most efficient and fast-responding *alc* system. For Ves2, the GUS protein expression level at 12 h after induction was 28.49 times that of the uninduced control, and 54.95 times at 24 h after induction. Its induction folds were much higher than those of Ves3 and Ves4. In terms of induction speed, a 28.49-fold increase was achieved from 0 h to 12 h, accounting for 51.8% of the 24 h induction amount, which was consistent with the induction speed of Ves1 (50.7%), but its induction fold was much lower than that of Ves1 (Table 3), making it an efficient and fast-responding *alc* system.

### 3.4. Analysis of Agronomic Traits and Physiological Indicators

**Figure 7:**
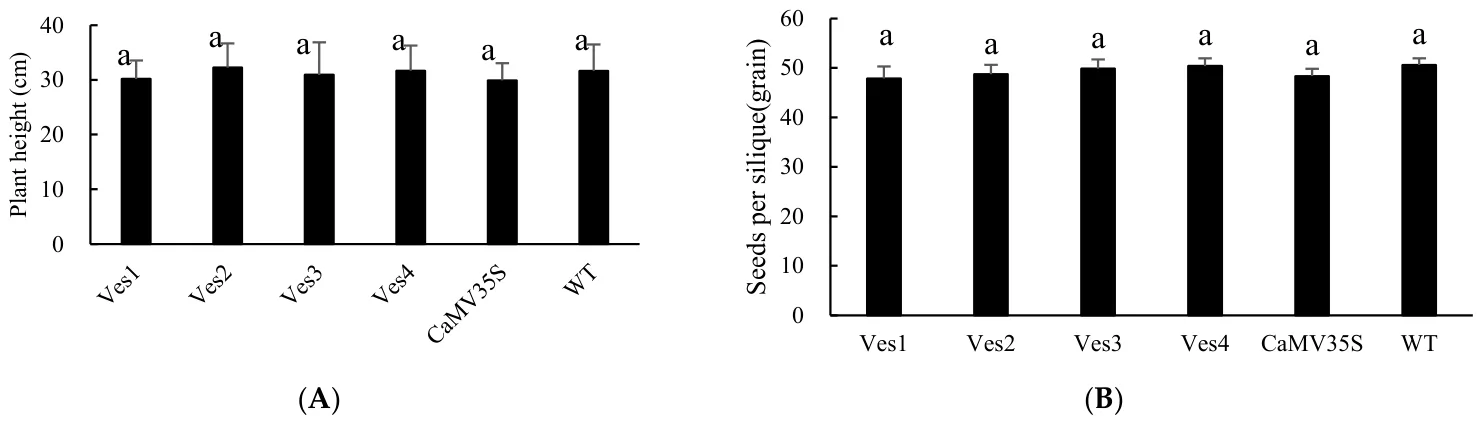
Plant heights (**A**) and seeds per silique (**B**) of transgenic *A. thaliana*. Three-week-old soil-grown wild-type plants, 35S-GUS plants, and four independent *alcA*-GUS plants were treated with 1.5% ethanol root drench for 48 h. When their seeds matured, plant height and seeds per silique were measured. Mean plant height and seeds per silique were shown for wild-type plants, 35S-GUS plants, and four independent *alcA*-GUS plants. Data are mean ± SD. The same letters in the same column chart indicate no significant differences (*p* > 0.05).

To investigate whether the concentration of ethanol used and the treatment duration affected plant growth and development, three-week-old soil-grown *A. thaliana* seedlings were treated with 1.5% ethanol root drench for 48 h. The physiological indicators of leaves and the agronomic traits of plants were then measured using an ultraviolet spectrophotometer (X-7S, Shanghai Yuanxi Instrument Co., Ltd., Shanghai, China) and a tape measure (Deli 2.0 m, Deli Group Co., Ltd., Ningbo, China), respectively. The results showed that the chlorophyll a, chlorophyll b, and total chlorophyll (a + b) content, SOD, POD, and CAT activities, and plant height and seeds per silique exhibited minimal variation. No significant differences were observed for these agronomic traits and physiological indicators among the five types of transgenic plants (Ves1, Ves2, Ves3, Ves4, and CaMV35S) and the wild-type plants (Table 4 and Table 5, and Figure 7). These results indicated that the treatment with 1.5% ethanol for 48 h had no effect on these physiological indices and agronomic traits of the plants.

## 4. Discussion

### 4.1. AlcR Binding Sites of the alc System

The design of the ethanol-inducible gene expression system was derived from *A*. *nidulans* [53,66]. In *A*. *nidulans*, the *alcR* gene encodes a specific activator (AlcR) of the ethanol utilization pathway. This activator regulates the expression of the *alcA* gene (encoding alcohol dehydrogenase I) and the *aldA* gene (encoding aldehyde dehydrogenase). In the absence of ethanol, the AlcR protein is inactive and cannot activate the expression of target genes. In the presence of ethanol, the AlcR protein is activated and binds to specific sites in the promoter of target genes, thereby inducing their expression [67,68]. To date, the working mechanism of the *alc* system and the specific promoter sequences bound by the AlcR protein have been well characterized, and the *alc* system has proven to be an important tool for analyzing gene function and for applications in plant biotechnology [24,54,69]. To develop an efficient *alc* system, we evaluated four different modifications of the *alcA* promoter. It was found that the tandem inverted repeat sequences bound by AlcR significantly increased the expression level of the *alc* system, indicating that this sequence possesses a unique ability to substantially enhance the *alc* system. Previous studies have shown that when the AlcR-binding sequence is positioned 240 bp upstream of the transcriptional start site, it is critical for ethanol-induced expression from the *alcA* promoter [62]. However, other studies have found that when the AlcR binding sites are located within 186 bp of the transcriptional start site, the *alc* system exhibits strong ethanol inducibility, suggesting that the exact positions of these binding sites are not crucial for ethanol-induced expression in plants [41]. We propose that the high efficiency of the *alc* system is associated with both the tandem inverted repeat sequences bound by AlcR and their positions. By optimizing the AlcR-binding inverted repeat sequences, integrating different copy numbers of these repeats, and simultaneously optimizing their positions within the *alcA* promoter, we are confident that a more efficient ethanol-inducible *alc* system can be developed. Ethanol root drench and vapor induction could effectively induce the *alc* system in *A. thaliana*, whereas foliar spray was relatively less effective. These results were supported by earlier studies in tobacco showing that a root drench and a vapor induction gave approximately 5-fold and 10-fold higher expression than an aerial spray in tobacco leaves, respectively [29]. The efficient *alc* system developed in this study will enable this important gene expression technology to be better applied in the fields of gene function and plant biotechnology.

### 4.2. Activation Method of the alc System

Currently, the main methods for inducing the *alc* system in plants include the root drench method, the foliar spray method, and the vapor method. Therefore, this study analyzed the *alc* system using these three methods.

For the root drench method, this study used 1.5% ethanol to analyze the performance of the constitutive expression system CaMV35S and four ethanol-inducible *alc* systems (Ves1–Ves4) in driving the expression of the *GUS* reporter gene. The results showed that the CaMV35S system was not affected by ethanol, whereas the *alc* system variants successfully achieved ethanol-inducible regulation. However, the induction efficiency varied significantly among the four *alc* systems (Ves1–Ves4), revealing differences in the effectiveness of their designs. Ves1 demonstrated excellent inducibility. At the early stage of induction (12 h), its GUS expression level had already reached a high level, showing no statistically significant difference from that of the strong constitutive promoter CaMV35S. As the induction time extended to 24 h and 48 h, the expression level of Ves1 not only significantly exceeded that of CaMV35S but also reached levels that were 1.36-fold and 3.34-fold those of the latter at 24 h and 48 h, respectively. Notably, the expression level of Ves1 in the late induction stage far surpassed that driven by the constitutive promoter, which is consistent with previous findings in plants such as tomato and sugarcane, where the *alc* system achieved expression levels comparable to or even higher than those of CaMV35S [41,52]. In contrast, the induction efficiencies of Ves2 and Ves3 were relatively low, being significantly weaker than that of the CaMV35S system at the early induction stages (12 h and 24 h). Although the expression level of Ves2 at 48 h significantly exceeded that of CaMV35S and Ves3 reached a high level with no statistically significant difference from the strong constitutive promoter CaMV35S, their overall induction kinetics were slower than those of Ves1. This indicates that, despite being based on the same *alc* regulatory principle, subtle differences in vector construction may significantly affect the binding efficiency of the transcription factor AlcR, chromatin accessibility, or interaction with the basal transcription machinery, ultimately leading to differences in induction strength and kinetics [12,41]. However, Ves4 consistently exhibited low-level expression throughout the experimental period, significantly lower than that of all other systems. This may suggest unexpected sequence issues or regulatory elements in the native *alcA* promoter (p*alcA*), resulting in a very weak response to ethanol induction signals—or even the presence of inhibitory structures—which makes this promoter unsuitable as an effective inducible expression tool. This also highlights the necessity of incorporating the 35S minimal promoter into the *alc* system [27,41,42].

For the foliar spray method, this study used 5.0% ethanol foliar spray as the induction method to evaluate the expression characteristics of the constitutive CaMV35S system and the ethanol-inducible *alc* system (Ves1–Ves4). The results indicated that ethanol foliar spray could effectively regulate the *alc* system but did not affect the activity of the CaMV35S system. However, the overall expression level of the *alc* system under foliar spray was significantly lower than that observed in the previous study using 1.5% ethanol root drench. This phenomenon is highly consistent with the previous findings in sugarcane, which clearly showed that root drench treatment effectively induced the expression of the modified *alc* system in sugarcane leaves and stems, whereas the spray method was relatively inefficient [41]. This finding has significant methodological implications: the induction efficiency of the *alc* system is closely related to the application method of the inducer. We found that ethanol root drench effectively induced target gene expression of the *alc* system in *Arabidopsis* leaves and floral organs. This induction may result from the combined effects of ethanol transported to the leaves and floral organs via xylem vessels and ethanol vapor released from the soil. This could also explain why root drench is more effective than foliar spray. Ves1 system performed optimally under root drench conditions, but under 5.0% ethanol foliar spray induction, its GUS expression levels did not surpass those of the CaMV35S system at any of the three time points. This may be related to the dose-dependent induction characteristics of the *alc* system. Previous studies in tobacco and *A. thaliana* showed that expression mediated by the *alc* system was dose-dependent, with extremely low background activity in the absence of an exogenous inducer [39,49]. Due to the short retention time and limited absorption efficiency of ethanol on the leaf surface, foliar spray fails to deliver an effective dose sufficient to fully activate the *alc* system, resulting in lower expression levels of the target gene. Another possible reason is that the induction efficiency of the *alc* system is affected by the transport and distribution of the inducer within the plant. Research has confirmed that the *alc* system can induce expression in both leaves and roots simultaneously through root drench or whole-plant treatment [29]. This suggests that ethanol transport within the plant is limited, so foliar spray may primarily affect the treated leaves, making it difficult to achieve uniform induction throughout the whole plant, thus leading to lower expression levels of the target gene. The GUS expression levels of Ves2, Ves3, and Ves4 at the three time points (12 h, 24 h, and 48 h) were significantly lower than those of the CaMV35S system, showing a performance gradient of Ves1 > Ves2 > Ves3 > Ves4. This clearly indicates that even under the same regulatory principle, the specific combination of different regulatory elements has a decisive impact on induction efficiency, and subtle sequence differences can lead to substantial variations in transcriptional activation efficiency [12,41]. Nevertheless, Ves1 still demonstrated significant superiority over Ves2, Ves3, and Ves4 under these conditions, with its expression level consistently being the highest among the *alc* systems and reaching an efficiency comparable to the CaMV35S system at 24 h. This further consolidates the status of Ves1 as an efficient and reliable configuration within the *alc* system.

For the vapor induction method, this study used 95% ethanol vapor fumigation as the induction method to analyze the performance of the constitutive expression system CaMV35S and four ethanol-inducible *alc* systems (Ves1–Ves4) in driving the expression of the *GUS* reporter gene. The results showed that ethanol vapor did not affect the activity of the constitutive CaMV35S system but could efficiently activate the *alc* systems. However, the induction efficiency varied significantly among *alc* systems, revealing differences in their design effectiveness. Among these, Ves1 demonstrated exceptionally inducible expression performance. Its expression level significantly surpassed that of the strong constitutive CaMV35S system as early as 12 h after induction, reaching 5.08 times that of CaMV35S at 48 h. This finding highlights the powerful efficacy of combining ethanol vapor induction with optimized vector design, providing an ultra-efficient and tightly regulatable gene expression tool for the field of plant biotechnology. Compared with the previous root drench study (reaching 3.34 times that of CaMV35S at 48 h) and the foliar spray study (at most comparable to CaMV35S), the expression level of Ves1 under ethanol vapor induction achieved a qualitative leap. This result is highly consistent with the previous findings in tobacco, potato, and oilseed rape, which clearly indicated that low-concentration ethanol vapor can efficiently induce the *alc* system, and exposing the whole plant or foliar parts to an ethanol vapor environment enables uniform induction in both leaves and roots [29], as also confirmed in *A. thaliana*, where the *alc* system responds more strongly to ethanol vapor than to soil drench with ethanol [39]. The *alc* system variants showed a clear performance gradient of Ves1 > Ves2 > Ves3 > Ves4. The expression levels of Ves2 and Ves3 at 12 h and 24 h were significantly lower than those of CaMV35S, but by 48 h, Ves2 was significantly higher than CaMV35S, while Ves3 showed no significant difference from CaMV35S. Ves4 maintained consistently low expression levels even after prolonged induction (24 h and 48 h). This gradient is consistent with the patterns observed in the root drench and foliar spray studies, further confirming the decisive influence of structural differences among different *alc* system variants on induction efficiency. This may be related to the fact that the induction efficiency of the *alc* system is comprehensively affected by multiple factors, including the structure of the *alcA* promoter region, the copy number of AlcR binding sites, and the minimal promoter sequence [12,41]. The positioning of Ves1 as “the most efficient chemical induction system” and Ves2 as “a highly efficient chemical induction system” provides a clear basis for selection in subsequent applied research.

### 4.3. Performance of the Different alc System

The research results once again confirmed the existence of a clear performance gradient within the *alc* system (Ves1 > Ves2 > Ves3 > Ves4). Ves1 demonstrated superior performance compared to the other *alc* systems under all three different ethanol induction methods (root drench, foliar spraying, and vapor fumigation), with its advantage being amplified to an extremely high level, especially under vapor induction. This strongly suggests that the vector configuration of Ves1 (such as specific promoter elements, enhancer sequences, or the arrangement of AlcR binding sites) has been carefully optimized, endowing it with extremely high sensitivity to ethanol signals and potent transcriptional driving capability, allowing it to be classified as the “most efficient” *alc* system. Ves2 showed expression significantly higher than CaMV35S after 48 h of root drench and vapor induction and can be categorized as a “highly efficient” *alc* system. After 48 h of ethanol root drench or vapor induction, Ves3 has no significant difference in expression level with CaMV35S, which can be classified as an effective *alc* system. In contrast, Ves4 exhibited poorer performance. This performance difference provides researchers with a diverse set of tools: Ves1 is the preferred choice when maximizing expression output is required; Ves2 or Ves3 may be more suitable when moderate intensity or gentler induction is desired. The consistently low expression level of Ves4 suggests potential defects in its construction and limited practical utility [41]. Future research could further explore the molecular mechanisms underlying the high expression efficiency of Ves1, delve into the induction characteristics of Ves1 in different plant species, evaluate its application effects in studying gene functions related to important agronomic traits and in crop improvement, and combine it with the latest gene editing technologies to develop more precise and efficient chemical-inducible expression systems. With a deeper understanding of the regulatory mechanism of the *alc* system, this chemical-inducible expression system is poised to play an even greater role in the fields of plant functional genomics and agricultural biotechnology.

## 5. Conclusions

In this study, an ethanol-inducible gene expression system (alcR/alcA) was developed through codon optimization of the *alcR* gene and *GUS* gene, integration of enhancer sequences and Kozak sequences upstream of these genes, and incorporation of varying numbers of specific binding sites for the AlcR protein into the *alcA* promoter. Using the 35S promoter as a reference, a comparative study of the *alc* system was conducted via ethanol root drench, foliar spray, and vapor application. The following clear conclusions were drawn: (1) The ethanol-inducible *alc* system was successfully constructed and validated for its operability in plants. Significant differences among various *alc* systems highlight the necessity of meticulous design and screening promoter structures during the development and application of *alc* inducible systems. (2) Among 1.5% root drench, 5.0% foliar spray, and 95% vapor induction, ethanol vapor induction was identified as the optimal method for achieving ultra-high expression levels of the *alc* system. Its induction effect far exceeded that of root drench and foliar spray with foliar spray being the least effective induction method. (3) Ves1 was identified as the most efficient chemical induction system, demonstrating outstanding induction efficiency. Ves2 was identified as a highly efficient chemical induction system, serving as an alternative option for high-efficiency induction.

## Figures and Tables

**Table 1:** Comparison of ethanol-inducible *alc* system expression in *A. thaliana* via root drench.

Plant	Induction Time			Ratio	
	0 h	12 h	24 h	12 h/0 h	24 h/0 h
Ves1	0.4375	24.2991	37.7278	55.54	86.23
Ves2	0.4315	10.0937	14.8398	23.39	34.39
Ves3	0.4162	8.9783	12.9945	21.57	31.22
Ves4	0.3949	1.9498	3.0494	4.94	7.72

**Table 2:** Comparison of ethanol-inducible *alc* system expression in *A. thaliana* via leaf spray.

Plant	Induction Time			Ratio	
	0 h	12 h	24 h	12 h/0 h	24 h/0 h
Ves1	0.3904	17.1952	26.6142	44.04	68.16
Ves2	0.3747	6.7292	10.4343	17.96	27.85
Ves3	0.3665	5.7519	8.2971	15.69	22.64
Ves4	0.3573	2.9059	3.8240	8.13	10.70

**Table 3:** Comparison of ethanol-vapor-inducible *alc* system expression in *A. thaliana*.

Plant	Induction Time			Ratio	
	0 h	12 h	24 h	12/0 h	24/0 h
Ves1	0.4255	40.6154	80.0552	95.45	188.14
Ves2	0.4064	11.5764	22.3327	28.49	54.95
Ves3	0.4074	8.2227	19.4651	20.18	47.78
Ves4	0.3992	2.6856	6.6645	6.73	16.69

**Table 4:** Chlorophyll content of the leaves of transgenic *A. thaliana*.

Plant	Chlorophyll a (mg/g)	Chlorophyll b (mg/g)	Chlorophyll (a + b) (mg/g)
Ves1	0.5295 ± 0.0084 a	0.2887 ± 0.0036 b	0.8183 ± 0.0120 c
Ves2	0.5121 ± 0.0335 a	0.2902 ± 0.0104 b	0.8023 ± 0.0233 c
Ves3	0.5570 ± 0.0271 a	0.2575 ± 0.0094 b	0.8144 ± 0.0279 c
Ves4	0.5207 ± 0.0142 a	0.2900 ± 0.0102 b	0.8107 ± 0.0240 c
Ves5	0.5434 ± 0.0480 a	0.2775 ± 0.0112 b	0.8209 ± 0.0391 c
WT	0.5485 ± 0.0443 a	0.2668 ± 0.0117 b	0.8154 ± 0.0367 c

Chlorophyll contents were measured in leaves using 3-week-old wild-type (WT) plants and transgenic plants containing a single T-DNA insertion of the 35S-GUS or *alcA*-GUS construct. Mean chlorophyll contents were shown for wild-type plants, 35S-GUS plants, and four independent *alcA*-GUS plants for 48 h after treatment with 1.5% ethanol root drench. Data are mean ± SD. The same letters in the same column indicate no significant differences (*p* > 0.05).

**Table 5:** POD, SOD, and CAT activities of transgenic *A. thaliana*.

Plant	POD Activities (ΔOD_470_.min^−1^. g^−1^ FW)	SOD Activities (Unit.g^−1^ FW)	CAT Activities (mg.min^−1^. g^−1^ FW)
Ves1	4.46 ± 0.85 a	105.68 ± 4.54 a	7.35 ± 0.11 a
Ves 2	4.40 ± 1.25 a	106.9 ± 3.45 a	7.32 ± 0.05 a
Ves 3	4.65 ± 0.99 a	108.7 ± 2.97 a	7.31 ± 0.07 a
Ves 4	4.37 ± 0.69 a	101.61 ± 4.82 a	7.30 ± 0.16 a
CaMV35 S	5.16 ± 1.39 a	107.18 ± 2.72 a	7.28 ± 0.05 a
WT	4.58 ± 2.60 a	101.46 ± 4.82 a	7.30 ± 0.12 a

POD, SOD, and CAT activities were measured in leaves using 3-week-old wild-type (WT) plants and transgenic plants containing a single T-DNA insertion of the 35S-GUS or *alcA*-GUS construct. Mean activities were shown for wild-type-plants, 35S-GUS plants, and four independent *alcA*-GUS plants for 48 h after treatment with 1.5% ethanol root drench. Data are mean ± SD. The same letters in the same column indicate no significant differences (*p* > 0.05).

## Data Availability

The data presented in this study are available from the corresponding authors upon reasonable request.

## Supplementary Materials

The following supporting information can be downloaded at https://www.mdpi.com/article/10.3390/genes17091142/s1: Table S1: Structure of the various *alc* gene switch promoters tested in *A. thaliana*; Table S2: Separation of self-pollinated progenies from transgenic plants (T0) and screening for homozygous plants.
