# Supplementary material for: Generation of a Highly Efficient and Chemically Inducible Gene Expression System
Source: Genes (Basel). 2026 Sep 18;17(9):1142. doi: 10.3390/genes17091142 (PMC13606033; doi:10.3390/genes17091142)
Supplement: Supplementary file 1 [file genes-17-01142-s001.zip › genes-4536335-supplementary.pdf]

Supplemental Material

Table S1. Structure of the various *alc* gene switch promoters tested in *Arabidopsis thaliana*.

Vector used	Description	Rationale
Ves1	Integrates a CaMV 35S minimal promoter (min35S), a TMV omega sequence, a Kozak sequence, and four tandem copies of the AlcR inverted repeat binding sites	Assess the effect of four tandem copies of the AlcR inverted repeat binding site on the *alc* system
Ves2	Integrates a CaMV 35S minimal promoter (min35S), a TMV omega sequence, a Kozak sequence, and two tandem copies of the AlcR inverted repeat binding sites	Assess the effect of two tandem copies of the AlcR inverted repeat binding site on the *alc* system
Ves3	Integrates a CaMV 35S minimal promoter (min35S), a TMV omega sequence, a Kozak sequence, and zero tandem copies of the AlcR inverted repeat binding sites	Assess the effect of zero tandem copies of the AlcR inverted repeat binding site on the *alc* system
Ves4	Original *alc* switch used in this study	Assess the effect of the CaMV 35S minimal promoter(min35S) on the alcR/alcA system
Ves5	Constitutive CaMV35S expression system	Assess expression level of the CaMV 35S promoter compared with the alcR/alcA system

Table S2. Separation of self-pollinated progenies from transgenic plants (T0) and screening for homozygous plants.

**Vector used**	**Plant No.**	**Total number of plants**	**Number of positive plants**	**Number of negative plants**	**Positive/ negative**	**Chi-square ($\chi 2$)**	**Probability (p)**	**Separation law (3:1)**	**Gene copy numbe r**	**Homozygote No.**
Ves1	L1	30	23	7	3.29	0.044	0.833	Conformity	1	L1-2-2
	L2	30	23	7	3.29	0.044	0.833	Conformity	1	L2-2-3
	L3	29	23	6	3.83	0.287	0.592	Conformity	1	L3-1-2
	L5	28	22	6	3.67	0.190	0.633	Conformity	1	L5-4-2
	L6	30	24	6	4.00	0.400	0.527	Conformity	1	L6-5-1
Ves2	L2	28	22	6	3.67	0.190	0.663	Conformity	1	L2-3-5
	L3	29	24	5	4.80	0.931	0.335	Conformity	1	L3-2-4
	L4	27	22	5	4.40	0.605	0.437	Conformity	1	L4-6-2
	L6	27	21	6	3.50	0.111	0.739	Conformity	1	L6-5-4
	L7	30	23	7	3.29	0.044	0.833	Conformity	1	L7-6-1
Ves3	L2	27	19	8	2.38	0.310	0.579	Conformity	1	L2-1-4
	L4	28	22	6	3.67	0.190	0.663	Conformity	1	L4-5-3
	L5	25	17	8	2.13	0.653	0.419	Conformity	1	L5-2-6
	L6	28	19	9	2.11	0.762	0.383	Conformity	1	L6-3-5
	L8	27	19	8	2.38	0.309	0.579	Conformity	1	L8-6-4
Ves4	L3	29	23	6	3.83	0.287	0.592	Conformity	1	L3-4-6
	L4	27	21	6	3.50	0.111	0.739	Conformity	1	L4-6-1
	L5	24	19	5	3.80	0.222	0.637	Conformity	1	L5-2-5
	L7	25	19	6	3.17	0.013	0.908	Conformity	1	L7-3-2
	L8	28	22	6	3.67	0.190	0.663	Conformity	1	L8-2-5
Ves5	L1	29	20	9	2.22	0.562	0.453	Conformity	1	L1-1-1
	L2	25	17	8	2.13	0.653	0.419	Conformity	1	L2-1-3
	L3	29	21	8	2.63	0.103	0.748	Conformity	1	L3-1-2
	L4	26	18	8	2.25	0.462	0.497	Conformity	1	L4-1-1
	L5	28	19	9	2.11	0.762	0.383	Conformity	1	L5-1-2
